# Atrial CARdiac Magnetic resonance imaging in patients with embolic stroke of unknown source without documented Atrial Fibrillation (CARM-AF): Study design and clinical protocol

**DOI:** 10.1016/j.hroo.2022.01.005

**Published:** 2022-01-20

**Authors:** Irum D. Kotadia, Robert O’Dowling, Akosua Aboagye, Iain Sim, Daniel O’Hare, José-Alonso Lemus-Solis, Caroline H. Roney, Marc Dweck, Amedeo Chiribiri, Sven Plein, Laszlo Sztriha, Paul Scott, James Harrison, Deborah Ramsay, Jonathan Birns, Peter Somerville, Ajay Bhalla, Steven Niederer, Mark O’Neill, Steven E. Williams

**Affiliations:** ∗ King’s College London, London, United Kingdom; † Guy’s and St Thomas’ NHS Foundation Trust, London, United Kingdom; ‡ Centre for Cardiovascular Science, The University of Edinburgh, Edinburgh, United Kingdom; § Biomedical Imaging Science Department, Leeds Institute of Cardiovascular and Metabolic Medicine, University of Leeds, Leeds, United Kingdom; ‖ King’s College Hospital, London, United Kingdom; ¶ Princess Royal University Hospital, London, United Kingdom

**Keywords:** Ischemic stroke, Embolic stroke of unknown source, Atrial fibrillation, Atrial cardiomyopathy, Cardiac magnetic resonance imaging, Electrocardiogram

## Abstract

**Background:**

Initiation of anticoagulation therapy in ischemic stroke patients is contingent on a clinical diagnosis of atrial fibrillation (AF). Results from previous studies suggest thromboembolic risk may predate clinical manifestations of AF. Early identification of this cohort of patients may allow early initiation of anticoagulation and reduce the risk of secondary stroke.

**Objective:**

This study aims to produce a substrate-based predictive model using cardiac magnetic resonance imaging (CMR) and baseline noninvasive electrocardiographic investigations to improve the identification of patients at risk of future thromboembolism.

**Methods:**

CARM-AF is a prospective, multicenter, observational cohort study. Ninety-two patients will be recruited following an embolic stroke of unknown source (ESUS) and undergo atrial CMR followed by insertion of an implantable loop recorder (ILR) as per routine clinical care within 3 months of index stroke. Remote ILR follow-up will be used to allocate patients to a study or control group determined by the presence or absence of AF as defined by ILR monitoring.

**Results:**

Baseline data collection, noninvasive electrocardiographic data analysis, and imaging postprocessing will be performed at the time of enrollment. Primary analysis will be performed following 12 months of continuous ILR monitoring, with interim and delayed analyses performed at 6 months and 2 and 3 years, respectively.

**Conclusion:**

The CARM-AF Study will use atrial structural and electrocardiographic metrics to identify patients with AF, or at high risk of developing AF, who may benefit from early initiation of anticoagulation.

**Key Findings**

▪ Undiagnosed atrial fibrillation is the underlying cause of a significant proportion of strokes originally classified as embolic stroke of unknown source (ESUS).
▪ Atrial fibrillation is associated with marked structural abnormalities of the left atrium, including dilatation, spherical remodeling, and atrial late gadolinium enhancement, which may be quantified using atrial cardiac magnetic resonance imaging.
▪ This study will test the hypothesis that identification of these structural abnormalities using atrial cardiac magnetic resonance imaging can identify the subset of ESUS patients at risk of atrial fibrillation using the gold standard of implantable loop recorder monitoring as the comparator.
▪ A poststroke model assessing atrial substrate predictive of developing atrial fibrillation may allow for earlier detection of patients at thromboembolic risk and allow earlier initiation of anticoagulation in these patients.

## Introduction

Atrial fibrillation (AF) is a major risk factor for ischemic stroke resulting in significant morbidity and mortality, but may not be identified by routine clinical monitoring during post-stroke care.^1^ It is estimated that 25% of embolic strokes of unknown source (ESUS) can be attributed to undiagnosed AF, with the risk of stroke recurrence in this cohort being high following the primary stroke event.^2^^,^^3^ Secondary stroke risk reduction in AF patients can be achieved by timely initiation of anticoagulation therapy.

Current guidelines for initiation of anticoagulation mandate electrocardiographic documentation of AF.^4^ Two landmark trials (EMBRACE and CRYSTAL-AF) provide evidence that increased AF detection rates are possible with prolonged ambulatory monitoring or following insertion of an implantable loop recorder (ILR).^5^^,^^6^ However, median AF detection time in real-world studies ranges from 40 days (interquartile range: 14–84 days) to 112 days (interquartile range: 35–293 days).^5^^,^^7^ With a risk reduction from 12% to 4% per year (hazard ratio 0.34; 95% CI 0.36–0.79) following the use of anticoagulation, delays in initiation of therapy are likely to have an impact on the incidence of secondary stroke.^8^ In addition, a requirement for prolonged monitoring is associated with an increased cost burden and need for further resources to accommodate follow-up.

A further limitation on the requirement of AF documentation is the discordance in temporality, biological gradient, and specificity between AF and ischemic stroke previously reported.^9^, ^10^, ^11^ Both the ASSERT and IMPACT trials enrolled patients with an implantable cardiac device prior to the index stroke event and demonstrated discordance in temporality, with up to 16% of patients in the ASSERT study being diagnosed with AF *after* the index stroke event despite prior device insertion.^9^, ^10^, ^11^ These studies suggest that elevated thromboembolic risk may exist prior to clinical detection of AF. Furthermore, while the ROCKET-AF Trial suggested an increase in adjusted rates of stroke or systemic embolism in the persistent AF group compared with the paroxysmal AF group (OR: 2.18 vs 1.73), the RE-LY Trial found no statistically significant change in thromboembolic risk between groups, suggesting that increased duration of AF may not be associated with increased stroke risk.^12^^,^^13^ In addition, previous meta-analyses have suggested that restoration of sinus rhythm does not reduce the risk of stroke,^14^^,^^15^ providing the underlying basis for continuation of anticoagulation for the purposes of stroke risk reduction regardless of treatment strategy following AF diagnosis if risk factors are present. As such, it is conceivable that patients with short paroxysms of AF who remain at high risk for secondary stroke may be undiagnosed by current standards of poststroke investigation of AF, which often include an electrocardiogram (ECG) and 24-hour Holter monitor. Combined, these studies suggest that elevated thromboembolic risk may exist prior to clinical detection of AF and represent a further delay in initiation of anticoagulation therapy in a high-risk cohort.

Previous ESUS studies, including NAVIGATE-ESUS and RESPECT-ESUS, have unsuccessfully attempted to identify this cohort for early initiation of anticoagulation.^16^^,^^17^ Recent models suggest comorbidities associated with elevated stroke risk (eg, hypertension, diabetes, vascular disease) and known to be associated with atrial disease may provide an atrial thromboembolic substrate even prior to the development of clinical arrhythmia.^9^^,^^18^

*A poststroke model assessing atrial substrate predictive of developing AF may allow for earlier detection of patients at thromboembolic risk, allowing for earlier initiation of anticoagulation.*

A novel term, “atrial cardiomyopathy,” has been described that refers to atrial remodeling resulting in “clinically relevant structural and electrophysiological changes of the atrium.”^19^ Atrial fibrosis plays a central role in the AF substrate, having been identified histologically in patients with AF and in patients with risk factors for AF. Atrial geometry also plays an important role in the pathogenesis of AF, with left atrial enlargement being greater in patients with persistent vs paroxysmal AF and atrial sphericity more pronounced in those with recurrent AF.^20^ In patients with AF, increasing atrial late gadolinium enhancement (LGE) on cardiac magnetic resonance (CMR) imaging is associated with increased risk of major adverse cardiovascular and cerebrovascular events, driven primarily by an increased risk of stroke or transient ischemic attack.^21^ Although atrial CMR imaging has frequently been used for substrate assessment in patients with known AF, few studies have published data regarding atrial substrate assessment in ESUS patients, highlighting a key knowledge gap.^22^ Identifying ESUS patients displaying these structural changes in the absence of AF may arguably identify patients at higher risk of developing AF and who may benefit from early anticoagulation poststroke.

Similarly, electrophysiological changes present on surface ECG have previously been highlighted in the literature as indirect surrogate markers of atrial cardiomyopathy and stroke. Analyses of p waves from the surface ECG show that p-wave terminal force in lead V_1_ (PTFV_1_) is independently associated with stroke, as well as structural atrial disease including atrial dilatation and fibrosis.^23^^,^^24^ Both PTFV_1_ and supraventricular ectopy have also previously been shown to be strong predictors of paroxysmal AF in ischemic stroke patients.^25^^,^^26^

Taken together, these observations suggest that left atrial structural and electrical remodeling are associated with stroke and predictive of the presence of paroxysmal AF even in the absence of documented AF at the time of the index stroke.

The CARM-AF study therefore seeks to detect atrial remodeling in a subset of patients with prior ischemic stroke and no AF detected during routine assessment. We hypothesize that the degree of structural and electrical remodeling present at the time of the first stroke will be predictive of the subsequent occurrence of AF as documented by gold-standard long-term ILR monitoring. The results of CARM-AF can inform future randomized controlled trials where clinical decisions regarding anticoagulation therapy are made at the time of the index stroke, potentially reducing the risk of secondary stroke.

## Objectives

The overall research aim is to produce a substrate-based predictive model using CMR and electrocardiographic parameters that may better identify ESUS patients at risk of developing AF.

The principal research question is to determine if atrial CMR imaging can predict the occurrence of AF in advance of the clinical arrhythmia in patients with confirmed ischemic stroke. The specific hypotheses that will be tested are as follows: (1) left atrial LGE in patients with ESUS is greater in patients with AF than those without AF documented by ILR; and (2) left atrial sphericity in patients with ESUS is greater in those patients with AF than those without AF documented by ILR.

The secondary research question is to determine whether electrocardiographic parameters can predict the occurrence of AF in advance of the clinical arrhythmia in patients with confirmed ischemic stroke. The specific hypotheses that will be tested are as follows: (3) PTFV_1_ in patients with ESUS is greater in those patients with AF than those without AF documented by ILR; and (4) supraventricular ectopy in patients with ESUS is greater in those patients with AF than those without AF documented by ILR.

As described in Data Analysis, below, further analysis will be performed to combine structural and electrical variables into a single score to improve prediction of AF status, which can be employed in future randomized controlled trials.

## Design

The CARM-AF Study is a multicenter, prospective, observational study of patients who have been diagnosed with an embolic stroke of unknown source in the absence of documented atrial fibrillation. The study conforms to the principles outlined in the Declaration of Helsinki. Ethical approval has been granted by Health Research Authorities and the South London Research Ethics Committee (REC: 19/LO/1933). The study is funded by the British Heart Foundation (PG/19/44/34368).

### Trial design

**Figure 1:**
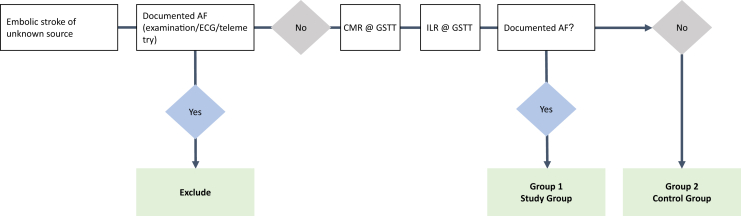
Trial flowchart. AF = atrial fibrillation; CMR = cardiac magnetic resonance; GSTT = Guy’s and St Thomas’ Hospital; ILR = implantable loop recorder.

A total of 92 patients will be recruited to undergo a CMR scan for investigation of features suggestive of left atrial remodeling. This will be followed by implantation of an ILR as standard of care for detection of AF as per the 2021 NICE guidelines.^27^ Remote ILR follow-up will occur at 6 months, 1 year, 2 years, and 3 years as part of this study. Throughout the study period routine follow-up will be conducted by daily telemetric monitoring using the Reveal CareLink system (Medtronic). Patients in whom AF has been detected will be allocated to the study group and patients in whom AF has not been detected will be allocated to the control group (Figure 1). Group allocation will be determined by 2 independent experts.

### Sample size justification

Previous data suggest that ILR monitoring will identify AF in 25% of ESUS patients with a CHA_2_DS_2_VaSc score of 3.^2^ We anticipate that our study population will have a 3:1 ratio of non-AF and AF patients. This ratio has subsequently been validated in the recent LOOP study.^28^

**Figure 2:**
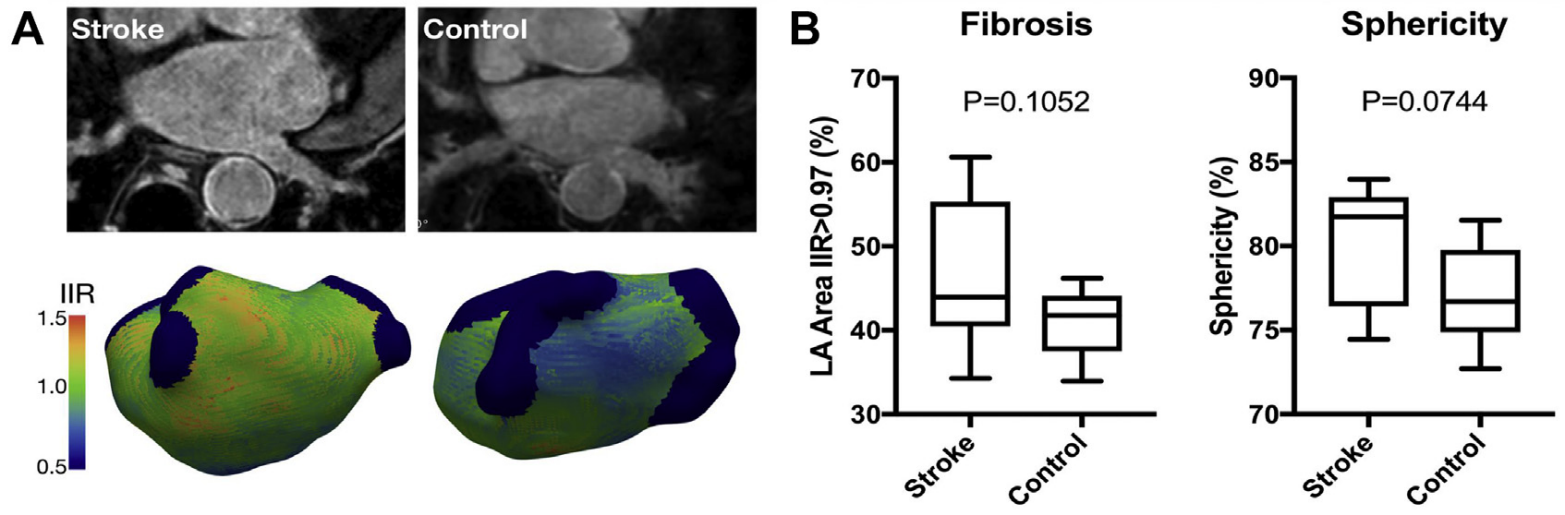
Atrial cardiac magnetic resonance (CMR) pilot data in stroke and control patients. Illustration of atrial CMR findings in stroke (n = 8) and control patients (n = 8). **A:** Raw data and processed data appearances. **B:** Late gadolinium enhancement and sphericity quantification illustrating medians, interquartile ranges, and data ranges. IIR = image intensity ratio; LA = left atrium.

Pilot data analysis was performed using CEMRGapp, using the image intensity ratio to define atrial fibrosis at image intensity ratio >0.97.^29^ This analysis suggested a population mean difference of 6% in left atrial LGE burden between AF and non-AF patients, with standard deviations of 9% and 4% in the AF and non-AF group, respectively (Figure 2). For estimating the sample size required to detect a difference in LGE burden between stroke patients with and without AF, we allow for unequal variances and assume that cases (AF on ILR monitoring) represent 25% of the study population while controls (no AF on ILR monitoring) represent 75% of the study population. Group sample sizes of 21 and 63 would achieve 80.8% power to reject the null hypothesis of equal means when the population mean difference is μ1 − μ2 = 47.0 − 41.0 = 6.0, with standard deviations of 9.0 for group 1 and 4.0 for group 2, and with a significance level (alpha) of 0.05 using a 2-sided 2-sample unequal-variance *t* test.

For estimating the sample size required to detect a difference in sphericity between stroke patients with and without AF, we again assume cases and controls represent 25% and 75% of the study population, respectively. Group sample sizes of 14 and 42 would achieve 82.5% power to reject the null hypothesis of equal means when the population mean difference is μ1 − μ2 = 80.0 − 77.0 = 3.0, with a standard deviation for both groups of 3.3 and with a significance level (alpha) of 0.05 using a 2-sided 2-sample equal-variance *t* test. Taking the larger of these estimates and allowing for 10% loss to follow-up indicates that a sample size of 92 patients is required for this study. With this sample size, assuming the 3:1 ratio of non-AF to AF individuals we would be able to estimate the area under the curve of a receiver operating characteristic (ROC) curve with 95% confidence interval width (upper-lower confidence limit) of between 0.21 and 0.28 for area-under-the-curve values between 0.85 and 0.65, respectively.

### Eligibility

Potential research participants who meet the eligibility criteria (Table 1) are identified on the stroke unit or in the transient ischemic attack outpatient clinic. All patients undergo a minimum set of diagnostic investigations to assess eligibility, including brain imaging confirming ischemic stroke (computed tomography / magnetic resonance imaging [MRI]), vascular imaging (head and neck), and a minimum of 24 hours of heart rhythm monitoring (telemetry/24-hour Holter). Patients in whom the stroke etiology is determined after study inclusion (for example, if a cause of stroke is identified via CMR imaging) will be excluded from final analysis.

**Table 1:** Inclusion and exclusion criteria

Inclusion criteria	Exclusion criteria
• Patient consent or assent can be obtained • Confirmed acute ischemic stroke with evidence on brain CT and/or MRI within 3 months of study enrollment • Ischemic stroke of unknown source with brain imaging suspicious for embolic etiology • Expected survival >12 months • At least 1 additional stroke risk factor (ie, CHA_2_DS_2_VaSc ≥3) • Sinus rhythm on 12-lead ECG, 24 hours of heart rhythm monitoring (telemetry/Holter), and a regular pulse on clinical examination • Above 18 years of age	• Unable to obtain patient consent or assent • History of atrial fibrillation • Atrial fibrillation detected on ECG and/or telemetry/Holter (AF duration of at least 30 seconds required for diagnosis), eGFR <30 mL/min • Indication for pacemaker/implantable cardioverter-defibrillator • Contraindication to undergo cardiac MRI (eg, severe claustrophobia, unable to lie flat for prolonged period, ferromagnetic implant) • Carotid stenosis >50% on duplex ultrasound associated with anterior circulation infarction • Vertebrobasilar stenosis >50% on CT/MR angiography associated with posterior circulation infarction • Single, isolated lacunar stroke with a corresponding lacunar infarct on brain CT/MRI • Specific etiology for cause of stroke (eg, arteritis, dissection, drug abuse)

AF = atrial fibrillation; CT = computed tomography; ECG = electrocardiogram; MR = magnetic resonance; MRI = magnetic resonance imaging.

### Consent

If eligible for recruitment, patients are informed about the study, and a Patient Information Sheet and Informed Consent Form is provided. Capacity to consent is assessed by the patient’s clinical team and should the patient be unable to provide written informed consent, study information is provided to a consultee (next of kin) and assent is subsequently obtained from the patient with the consultee acting as a patient advocate. Patients are recruited via both methods in order to preserve the heterogeneity of the study population.

### Baseline data collection

On enrollment, patient data collected includes patient demographics, preexisting medical history, CHA_2_DS_2_VaSc score, and data from relevant stroke investigations (routine blood panel, carotid Dopplers, computed tomography / MRI brain).

### Noninvasive electrophysiological data

All patients will undergo a 12-lead surface ECG and 24 hours of heart rhythm monitoring using telemetry or 24-hour Holter prior to insertion of an ILR (LINQ; Medtronic, Mounds View, MN). Electrocardiographic metrics to be assessed include mean heart rate, atrial high rate episodes, atrial ectopy burden, PTFV_1_, and PR interval.

### Cardiac magnetic resonance imaging

CMR imaging is performed within 3 months of the index stroke and prior to ILR implantation. All imaging is performed on a 1.5 Tesla clinical MRI scanner. Localizer scans are acquired in the 3 standard imaging planes (sagittal, coronal, and transverse), followed by vertical long-axis, horizontal long-axis, and short-axis imaging. Cine imaging of the 4-chamber and 2-chamber views are acquired in end-expiration using a standard balanced steady-state free presession cine technique.

Gadolinium (Gadovist; Bayer HealthCare Pharmaceuticals, Berlin, Germany) is administered at 0.2 mmol/kg at a rate of 0.3 mL/s followed by 30 mL normal saline flush. Following gadolinium, an ECG-triggered, contrast-enhanced magnetic resonance angiogram (CE-MRA) 3D dataset is acquired to delineate the left atrial endocardial border 90 seconds after gadolinium administration, after which a full short-axis stack including the ventricles and atria is acquired.

For LGE imaging, the inversion time is determined from a multiphase inversion time mapping sequence (TI-Scout/Look-locker) performed immediately prior to each LGE scan to ensure adequate nulling of ventricular myocardium. Two separate sequences are used to acquire LGE imaging: a respiratory-navigated sequence and an image-navigated sequence. Respiratory navigator artefacts are a limitation of left atrial imaging that the image-navigated sequence seeks to eliminate.

For image-navigated imaging, an ECG-triggered free-breathing image-navigated dual-echo 3D inversion recovery spoiled gradient echo sequence (iNav LGE) is acquired 15–20 minutes after gadolinium administration according to our previous sequence optimization and routine clinical practice for preablation atrial imaging at our institution.^30^ The 2D image navigators enable data collection throughout respiration, ensuring no data rejection and significantly reducing scan time and, subsequently, motion artefact. Dual echo readout allows for water and fat separation, providing LGE images of both fat suppression and fat distribution.^31^ Coverage includes the left and right atrium. Typical parameters are as follows: TR 7.16 ms, TE 2.38/4.76 ms, flip angle 20°, centric k-space ordering, 1.3 × 1.3 × 4 mm^3^ acquired voxel size with interpolation to 1.3 × 1.3 × 2 mm^3^ during reconstruction, SPIR fat suppression, anterior-posterior phase encoding direction.

For respiratory-navigated imaging, a second LGE sequence is performed 20–25 minutes after gadolinium enhancement. This comprises an ECG-triggered free-breathing diaphragmatic navigated 3D inversion recovery spoiled gradient echo sequence (dNav LGE) that conforms to current clinical standards within our institution for atrial imaging. Coverage includes the left and right atrium. Typical parameters are as follows: TR 4.2 ms, TE 1.8 ms, flip angle 20°, centric k-space ordering, 1.3 × 1.3 × 4 mm^3^ acquired voxel size with interpolation to 1.3 × 1.3 × 2 mm^3^ during image reconstruction, SPIR fat suppression, anterior-posterior phase encoding direction, navigator gating acceptance window of 5 mm in end-expiration.

For all 3 of the 3D sequences (CE-MRA, iNav LGE, dNav LGE) subject-specific trigger delay and acquisition window are identified using the 4-Chamber cine image to identify atrial diastole and reduce cardiac motion artefact. The field of view is identical in both LGEs and the CE-MRA to facilitate registration between the images during postprocessing.

Primary structural metrics collected from CMR imaging include left atrial surface area and volume, visual and quantitative LGE assessment, and sphericity. Secondary anatomical metrics collected include left atrial appendage orifice size and flow.

### Image postprocessing

All CMR scans are postprocessed shortly after acquisition prior to completion of ILR follow-up, patient group assignment, and, where relevant, exclusion of patients owing to the identification of a secondary cause of stroke on CMR imaging (see Eligibility, above). This approach is taken in order to eliminate any potential postprocessing bias after study/control group allocation. Postprocessing is performed using the previously validated open-source Cardiac Electro-Mechanics Research Group App (CEMRG) platform (http://www.cemrg.co.uk).^32^ Imaging is reinterpolated to 1 × 1 × 1 mm^3^. A total of 8–12 semiautomatic axial segmentations of the atrial blood pool are then performed using the CE-MRA image and interpolated to reconstruct a 3D atrial shell. Rigid body registration is then used to align the CE-MRA and dNav LGE image. This is performed by identifying the rotations and translations required to optimize the normalized mutual information as the similarity measure of the 2 images giving rise to a displacement field. This is then used to transform the CE-MRA segmentation and overlay it with the dNav LGE image for review of image alignment. In case of malalignment, segmentation is performed using the dNav image directly.

Next, the pulmonary veins and appendage are removed from the atrial shell. The distal end of each pulmonary vein and the left atrial appendage are manually tagged. Center lines are formed from these points and converge to a central point in the body of the left atrium. Pulmonary vein/appendage clippers are then displayed perpendicular to the entry of the vein/appendage into the body of the left atrium. This is defined by the rate of increase of the cross-section area of the vein/appendage, starting from the distal end of the vein/appendage and toward the atrial body. As the cross-section approaches the atrial body, its area increases substantially; this inflection is used to identify the opening of the chamber. Clippers can be manually adjusted prior to removal of the pulmonary veins/atrial appendage.

The last step prior to analysis is clipping of the mitral valve. In order to provide a clipping plane, the valve is viewed en face and 3 points around the valve are selected at 3, 7, and 10 o’clock to create a best-fit circle that is subsequently used as the orthodrome of a spherical clipper. The clipper is then manually reviewed for accuracy of valve positioning prior to valve removal.

#### Fibrosis assessment

The dNav LGE image is then interrogated and an LGE map generated using a maximum-intensity projection technique whereby a 3 mm external projection and 1 mm internal projection is used by default to adequately capture the full thickness of the atrial wall and to account for minor misalignment between the CE-MRA and LGE imaging. To quantify LGE, a blood pool segmentation is derived using a 3-voxel size erosion of the previously segmented left atrial body. Mean and standard deviation of blood pool signal intensity are automatically calculated and are used as reference values. LGE assessment is then performed using the image intensity ratio method using a range of predefined thresholds.^33^ ROC analysis will be performed to delineate the optimal image intensity ratio for use within the predictive model for identification of study vs control group allocation.

#### Sphericity assessment

Atrial sphericity is automatically calculated using the left atrial sphericity predictor method.^20^ A best-fit sphere of the left atrium is calculated using the center of mass determined by the average radius of all left atrial wall points. The coefficient of variation of the sphere is then used to identify the best-fit sphere. The deviation of the segmented atrial body from the best-fit sphere is then calculated to quantify sphericity.

#### Additional postprocessing for assessment of iNav LGE imaging

**Figure 3:**
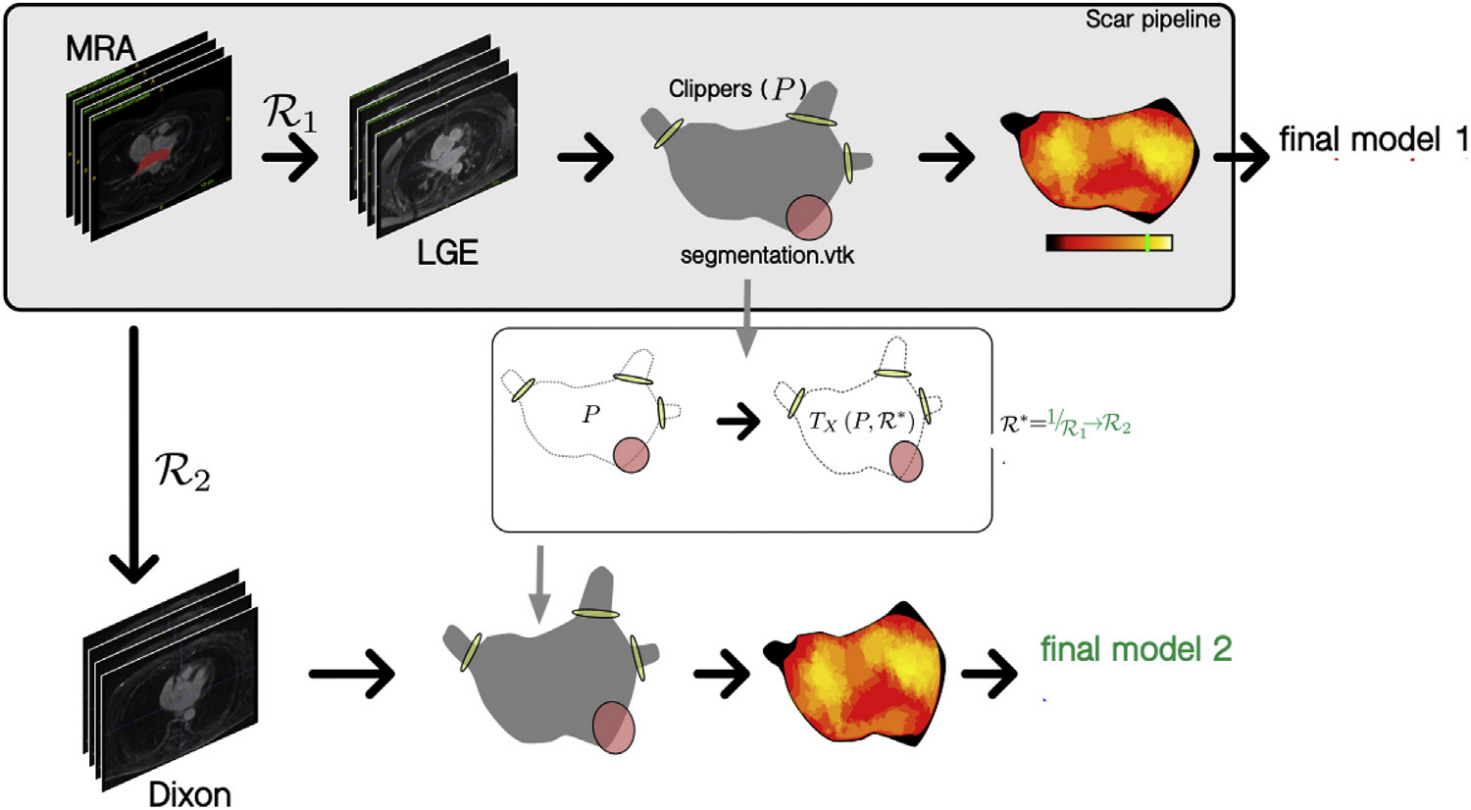
Work flow for postprocessing of image-navigated late gadolinium enhancement (LGE) imaging. MRA = magnetic resonance angiography; R = registration.

An additional registration step is performed for alignment of the CE-MRA atrial segmentation and iNav LGE image. Pulmonary vein, left atrial appendage, and mitral valve clipping planes are registered and transformed from the dNav LGE image and overlaid to the iNav LGE image using the same image registration and transformation techniques described earlier. An LGE map can then be created and LGE and sphericity quantified as performed for dNav LGE imaging for comparison (Figure 3).

#### Implantable loop recorder programming

All patients receive a Reveal LINQ (Medtronic) ILR device as per routine clinical care for detection of AF. Nominals are set to detect increased sensitivity of AF detection threshold, aggressive ectopy rejection, and storage of all AF/atrial tachycardia episodes to maximize AF detection. AF is detected following 2 minutes of ILR monitoring as per the Medtronic AF detection algorithm. Each episode will be reviewed by 2 independent experts prior to study/control group allocation. Daily remote data collection will occur as part of routine clinical care.

## Data analysis

Primary analysis will be performed following 12 months of continuous ILR monitoring with interim and delayed analyses performed at 6 months and 2 and 3 years, respectively. Descriptive statistics will be used to characterize the study population. Structural and electrocardiographic metrics will be summarized. Results will be reported as a comparison between the standard deviation and mean difference. If groups show a normal distribution, *t* test will be performed to assess for a significant difference between groups; otherwise, a Mann-Whitney *U* test will be used. The χ^2^ test will be used for categorical data. Any *P* value <.05 will be considered significant. For qualitative assessment of LGE, patients will be categorized as per our routine clinical practice using combined assessment of both LGE methods as none, mild, moderate, or severe LGE. Survival analysis will be performed using Kaplan-Meier curves with atrial fibrillation as the outcome measure. For qualitative assessment of atrial LGE, our open-source platform, CEMRGApp, will be used to create LGE scores. ROC analysis will be used to determine if there is a quantitative LGE cut-off with adequate specificity and sensitivity to detect atrial fibrillation during follow-up. To combine these variables into a single score, the use of logistic regression, shallow classifiers, atrial computational modeling, or combining variables using summated z-scores will be explored to improve the predictive value for AF.

## Trial significance

At present, there is limited published data regarding advanced atrial CMR imaging in either non-AF patients or ESUS patients. The CARM-AF Study will provide novel information regarding the difference in structural and electrical remodeling in patients with and without AF. Several large randomized controlled trials including CRYSTAL-AF and ROCKET-AF provide evidence to support prolonged heart rhythm monitoring in ESUS patients. The most recent 2021 NICE guidelines advocate the use of ILR implantation as a diagnostic aid to improve AF detection but continues to rely on a diagnosis of AF for initiation of anticoagulation in the ESUS population. We aim to produce a substrate-based model that can be used to predict ESUS patients at higher risk of AF, allowing for initiation of anticoagulation at the time of stroke, rather than relying on future clinical arrhythmia detection. Furthermore, this would negate the need for ILR insertion and follow-up, resulting in a significant reduction in cost burden and use of limited resources.

We anticipate that this study will form the basis of a randomized controlled trial with the potential to revolutionize poststroke care in ESUS patients, where patients are anticoagulated on the basis of atrial substrate rather than the presence or absence of an AF clinical diagnosis.
